# Influence of social lifestyles on host–microbe symbioses in the bees

**DOI:** 10.1002/ece3.10679

**Published:** 2023-11-02

**Authors:** Lauren Mee, Seth M. Barribeau

**Affiliations:** ^1^ Institute of Infection, Veterinary and Ecological Sciences, Department of Evolution, Ecology and Behaviour University of Liverpool Liverpool UK

**Keywords:** bee, coevolution, microbiome, mNGS, sociality, symbionts

## Abstract

Microbiomes are increasingly recognised as critical for the health of an organism. In eusocial insect societies, frequent social interactions allow for high‐fidelity transmission of microbes across generations, leading to closer host–microbe coevolution. The microbial communities of bees with other social lifestyles are less studied, and few comparisons have been made between taxa that vary in social structure. To address this gap, we leveraged a cloud‐computing resource and publicly available transcriptomic data to conduct a survey of microbial diversity in bee samples from a variety of social lifestyles and taxa. We consistently recover the core microbes of well‐studied corbiculate bees, supporting this method's ability to accurately characterise microbial communities. We find that the bacterial communities of bees are influenced by host location, phylogeny and social lifestyle, although no clear effect was found for fungal or viral microbial communities. Bee genera with more complex societies tend to harbour more diverse microbes, with *Wolbachia* detected more commonly in solitary tribes. We present a description of the microbiota of Euglossine bees and find that they do not share the “corbiculate core” microbiome. Notably, we find that bacteria with known anti‐pathogenic properties are present across social bee genera, suggesting that symbioses that enhance host immunity are important with higher sociality. Our approach provides an inexpensive means of exploring microbiomes of a given taxa and identifying avenues for further research. These findings contribute to our understanding of the relationships between bees and their associated microbial communities, highlighting the importance of considering microbiome dynamics in investigations of bee health.

## Graphical abstract

Microbiome diversity in bees from various social lifestyles and taxa was investigated using cloud computing and publicly available transcriptomic data. Results show that bacterial communities in bees are influenced by host location, phylogeny, and social lifestyle, with more complex bee societies hosting more diverse microbes. The presence of bacteria with anti‐pathogenic properties across social bee genera suggests the importance of symbiotic relationships in enhancing host immunity, contributing to our understanding of the interplay between bees and their associated microbial communities and emphasising the significance of microbiome dynamics in bee health research.

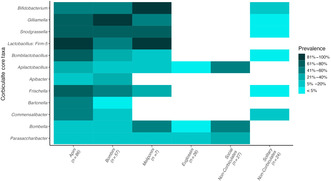

## 1 INTRODUCTION

In the insect world, microbial symbionts can play a major role in many biological processes (Munoz‐Benavent et al., 2021), including reproduction (Bourtzis et al., 1996; Singh & Linksvayer, 2020; Werren et al., 2008), nutrition (Andersen et al., 2012; Cheng et al., 2019) and pathogen defence (Benoit et al., 2017; Bian et al., 2010; Duplouy et al., 2015). For social insects, where consistent social contact between conspecifics allows for high‐fidelity vertical transmission of microbial communities, these symbionts can be passed on for generations, allowing for coevolution of microbiome and host (Dietrich et al., 2014; Kwong, Medina, et al., 2017; Lombardo, 2008; Sanders et al., 2014; Zhang & Zheng, 2022). This has been demonstrated in the obligately eusocial corbiculate bees, which all share a core set of bacterial microbes (Koch et al., 2013; Koch & Schmid‐Hempel, 2011; Kwong, Medina, et al., 2017; Kwong & Moran, 2016; Lim et al., 2015; Moran et al., 2012). The members of this conserved bacterial community are important for the health of their hosts, particularly by protecting against infectious disease (Anderson et al., 2014; Koch & Schmid‐Hempel, 2012; Miller et al., 2021; Vásquez et al., 2012). However, there are very few bee microbial studies outside of these eusocial corbiculates (Handy et al., 2022; Kapheim et al., 2021; McFrederick et al., 2012, 2014, 2017; Rubin et al., 2018), meaning the microbiomes of the majority of bee species remain a mystery.

One of the current approaches of characterising the microbiome of a host is to use metagenomic Next‐Generation Sequencing (mNGS), where all DNA (or RNA) from a given environment, such as an insect gut, is sequenced and the microbial community characterised. While the cost of producing NGS data has dramatically reduced over recent years, it remains reasonably expensive, taking into account sample extraction, library production, sequencing costs and having the appropriate informatics infrastructure in order to store, process and analyse data (Krampis & Wultsch, 2015). One attractive solution for some analyses is to use cloud‐computing resources (Krampis & Wultsch, 2015). CZID.org, for example, is an approachable, open‐source cloud‐based service, which can provide microbial identification for many different sample types and host species (Kalantar et al., 2020).

**FIGURE 1:**
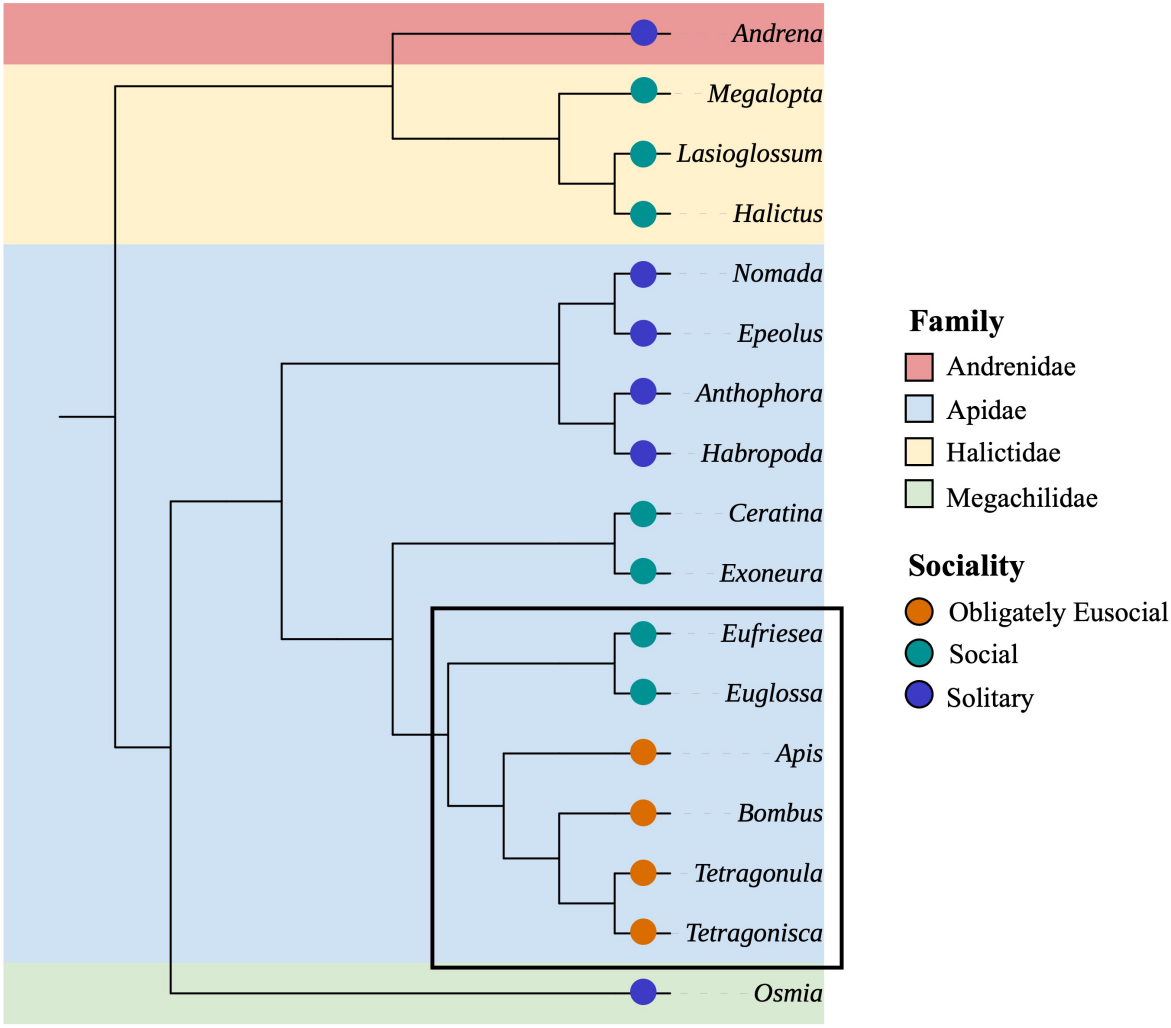
Cladogram of the genera included in these analyses coloured by family, with the sociality of each genus specified by a coloured circle. The corbiculate bees are marked within a black lined box. This tree is based on accepted topology in the literature (Bossert et al., 2019; Gibbs et al., 2012; Husemann et al., 2021; Jack, 2021; Kapheim et al., 2019; Lu et al., 2021). Branch lengths are not indicative of evolutionary time.

Here, we use this approach to examine NGS datasets from 18 bee genera spanning 100 million years of divergence (Figure 1, Bossert et al., 2019; Jack, 2021; Gibbs et al., 2012; Husemann et al., 2021; Kapheim et al., 2019; Lu et al., 2021; Peters et al., 2017) that vary in their social structure, ranging from solitary to obligately eusocial. We decided to test and see whether publicly available transcriptomic datasets generated for other purposes elucidate the microbiome of various bee taxa. To examine how the microbiome differs among social structure, we simplified the many different distinctions in social structure found in the literature to: (1) solitary, where species do not provide any brood care and associate with conspecifics only for mating; (2) social, which included any species that had considerable contact with conspecifics (i.e. communal nesting) and some brood care (primitively or facultatively eusocial) but where individuals can and do live solitarily; and (3) obligately eusocial species that only ever exhibit eusocial behaviours and solitary living is impossible (Figure 1). We used this framework to systematically test whether social structure, location or bee taxa affect microbial composition across the bees.

## 2 MATERIALS AND METHODS

### 2.1 Sample selection

We analysed sequence data sourced from NCBI's Sequence Reads Archive (SRA) (Katz et al., 2022; Kodama et al., 2012; Leinonen et al., 2010), accessed September 2022. We included all available RNA‐Seq adult bee samples that included the animal's abdomen (including pooled individuals), and we excluded projects that exclusively sequenced any other part (e.g. antennae, brain, ovaries), or developmental stages (e.g. larvae, pupae). We only included unaltered control specimens (i.e. no treatment or stressor introduced/administered) to ensure that the microbial composition was as natural as possible. Suitability was determined from metadata provided with the SRA sequencing data and any associated publications. SRA projects that had ambiguous, unclear or missing metadata were excluded from consideration.

### 2.2 Processing, mapping and uploading reads

All sequence data (fastq format, Table 1; Table S1) were downloaded and unpacked from the SRA using prefetch and fasterq‐dump from the SRA‐toolkit (version 3.0.0, Katz et al., 2022; Kodama et al., 2012; Leinonen et al., 2010). From here, we split the pipeline: sequencing data from the European honeybee *Apis mellifera* were uploaded directly to CZID.org using the command‐line interface (version 4.1.2), and non‐*A. mellifera* sequences were retained for further processing. CZID (Chan Zuckerberg ID, previously known as IDSeq, Kalantar et al., 2020) is a cloud‐based, open‐source platform that maps input sequence files against a chosen species genome and then aligns any unmapped reads to NCBI databases in order to detect non‐host sequences.

**TABLE 1:** List of host species with associated NCBI projects and references when available.

Tribe	Species	*n*	Project(s)	Reference(s)
Allodapini	*Exoneura* spp.	1	PRJNA687066	Brettell et al. (2020)
Andrenini	*Andrena* spp.	4	PRJNA687318	Daughenbaugh et al. (2021)
	*Andrena camellia*	4	PRJNA510543	
	*Andrena cineraria*	1	PRJNA411946	Schoonvaere et al. (2018)
	*Andrena fulva*	1	PRJNA411946	Schoonvaere et al. (2018)
	*Andrena haemorrhoa*	2	PRJNA411946	Schoonvaere et al. (2018)
	*Andrena vaga*	1	PRJNA318490	Schoonvaere et al. (2016)
Anthophorini	*Anthophora plumipes*	1	PRJNA252326	Peters et al. (2017)
	*Habropoda laboriosa*	1	PRJNA279436	Kapheim et al. (2015)
Apini	*Apis cerana*	5	PRJNA235974, PRJNA562784	Fan et al. (2022); Park et al. (2015)
	*Apis mellifera*	87	PRJNA274674, PRJNA357165, PRJNA357523, PRJNA380316, PRJNA495845, PRJNA510543, PRJNA531527, PRJNA681941, PRJNA687066, PRJNA754836, PRJNA793424, PRJNA820512	Brettell et al. (2019); Daughenbaugh et al. (2021); Galbraith et al. (2015); Lester et al. (2022); Melicher et al. (2019); Remnant et al. (2017); Roberts et al. (2017); Wang et al. (2021); Wu et al. (2017)
Augochlorini	*Megalopta genalis*	22	PRJNA331103	Jones et al. (2017)
Bombini	*Bombus* spp.	1	PRJNA704259	Pascall et al. (2021)
	*Bombus breviceps*	1	PRJNA659133	Sun et al. (2021)
	*Bombus confusus*	1	PRJNA659133	Sun et al. (2021)
	*Bombus consobrinus*	1	PRJNA659133	Sun et al. (2021)
	*Bombus difficillimus*	1	PRJNA659133	Sun et al. (2021)
	*Bombus haemorrhoidalis*	1	PRJNA659133	Sun et al. (2021)
	*Bombus ignitus*	1	PRJNA659133	Sun et al. (2021)
	*Bombus lucorum*	2	PRJNA704259	Pascall et al. (2021)
	*Bombus opulentus*	1	PRJNA659133	Sun et al. (2021)
	*Bombus pascuorum*	9	PRJEB43529, PRJNA318490, PRJNA411946, PRJNA704259, PRJNA659133	Darwin Tree of Life Project Consortium (2022); Pascall et al. (2021); Schoonvaere et al. (2016, 2018); Sun et al. (2021)
	*Bombus pyrosoma*	7	PRJNA646593, PRJNA646602, PRJNA646687, PRJNA646806, PRJNA646816, PRJNA646831, PRJNA659133	Liu et al. (2020); Sun et al. (2021)
	*Bombus rupestris*	1	PRJNA252285	Peters et al. (2017)
	*Bombus sibiricus*	1	PRJNA659133	Peters et al. (2017)
	*Bombus soroeensis*	1	PRJNA659133	Sun et al. (2021)
	*Bombus superbus*	1	PRJNA659133	Sun et al. (2021)
	*Bombus terrestris*	20	PRJNA295976, PRJNA318490, PRJNA411946, PRJNA615177, PRJNA704259	Amsalem et al. (2015); Araujo and Arias (2021); Pascall et al. (2021); Schoonvaere et al. (2016, 2018)
	*Bombus terricola*	12	PRJNA730495	Tsvetkov et al. (2021)
	*Bombus turneri*	1	PRJNA659133	Sun et al. (2021)
	*Bombus waltoni*	1	PRJNA659133	Sun et al. (2021)
Ceratinini	*Ceratina australensis*	5	PRJNA302035	Rehan et al. (2018)
Epeolini	*Epeolus variegatus*	1	PRJNA252262	Peters et al. (2017)
Euglossini	*Eufriesea mexicana*	1	PRJNA279814	Kapheim et al. (2015)
	*Euglossa dilemma*	7	PRJNA252310, PRJNA636137	Peters et al. (2017); Séguret et al. (2021)
	*Euglossa viridissima*	20	PRJNA636137	Séguret et al. (2021)
Halictini	*Halictus sexcinctus*	1	PRJNA374528	Ballenghien et al. (2017)
	*Lasioglossum* spp.	2	PRJNA687066	Brettell et al. (2020)
	*Nomada lathburiana*	1	PRJNA252330	Peters et al. (2017)
Meliponini	*Tetragonisca angustula*	6	PRJNA615177	Araujo and Arias (2021)
	*Tetragonula carbonaria*	2	PRJNA687066	Brettell et al. (2019)
Nomadini	*Nomada lathburiana*	1	PRJNA252330	Peters et al. (2017)
Osmiini	*Osmia bicornis*	8	PRJNA285788, PRJNA411946	Beadle et al. (2019); Schoonvaere et al. (2018)
	*Osmia cornuta*	4	PRJNA318490, PRJNA411946	Schoonvaere et al. (2016, 2018)
Rophitini	*Dufourea novaeangliae*	1	PRJNA279825	Kapheim et al. (2015)

*Note*: Tribe cells are coloured according to host family: blue for Apidae, red for Andrenidae, yellow for Halictidae, green for Megachilidae. All samples were assessed for presence of microbes, but not all samples were included in later analyses (i.e. beta diversity). See Table S1 for further details.

Briefly, the CZID pipeline (Kalantar et al., 2020) used in this analysis can be summarised in the following steps. Firstly, a genome and blank sample is chosen. The former is used to map input reads against, the latter is used to calculate the likelihood of alignment hits occurring due to contamination. The input sequences are validated before the first round of mapping reads against the chosen host genome (using STAR, Dobin et al., 2013; Dobin & Gingeras, 2016). The resultant unmapped reads are then processed to remove adaptor sequences, duplicated or low‐quality reads. These reads are then mapped again using a different genome mapper and, finally, unmapped reads are sub‐sampled and remaining reads are aligned against the NCBI nucleotide (NT) and non‐redundant protein (NR) sequence databases to identify the likely taxonomic source. The pipeline output is a CZID taxon report with all non‐host taxa hits and accompanying measurements, such as number of aligned reads, e‐values and z‐scores (used to determine likelihood of a read being contamination). In each of these non‐host taxa “hits,” the number of reads is recorded and these counts can be considered as representative of microbial transcriptional activity and therefore taxa presence and abundance.

The genome that original input sequences are mapped against is selected from a pre‐determined list, and at the time of the analysis (October 2022), the host genome option “Bee” included only the honeybee, *A. mellifera*, genome. Therefore, non‐*A. mellifera* samples required a number of pre‐processing steps. First, each sample was assigned the phylogenetically closest reference genome (see Table S2). Sample sequence files were then mapped against each respective genome using STAR (version 2.7.10a, Dobin et al., 2013; Dobin & Gingeras, 2016), the same genome mapper used as the first mapping step of CZID's pipeline (version 7.1). Every sample that achieved >50% of reads successfully mapping to the reference genome proceeded to the next step. For the samples that had ≤50% reads fail to map because they were “too short,” an indication of reads of various lengths not mapping well, we repeated the mapping with slightly relaxed parameters (‐‐outFilterScoreMinOverLread 0.3 ‐‐outFilterMatchNminOverLread 0.3). This was needed when the species was comparatively phylogenetically distant from the nearest available genome. Regardless of the success of the second mapping run, all unmapped sequence files were then uploaded to CZID.org for taxonomic assignment using pipeline version 7.1 as further parameter relaxation was deemed counter‐productive.

### 2.3 Taxonomy

All taxonomic classifications of the identified microbes were sourced from the NCBI taxonomy (taxonomy dump file from NCBI ftp service, Federhen, 2012; Schoch et al., 2020, accessed 18th October 2022). A single manual change was made: to distinguish the *Lactobacillus: Firm‐5* (also known as *Lactobacillus* near *melliventris*) as a separate genus to *Lactobacillus*, as this taxonomic cluster has repeatedly been found to be an important member of the corbiculate bee microbiome (Kwong, Medina, et al., 2017; Martinson et al., 2011; Vásquez et al., 2012). These species include *Lactobacillus apis*, *L. melliventris*, *L. kimbladii*, *L. kullabergensis*, *L. panisapium*, *L. bombicola* and *L. helsingborgensis* (Heo et al., 2020).

CZID also uses the NCBI taxonomy as the basis of its taxon reports, but, as it is only updated periodically, there were some minor differences between taxa identified as hits by CZID and corresponding classifications in the NCBI taxonomy dump file. In these instances, we updated the taxon reports to reflect the more recent classifications (NCBI). For all analyses, we only used genus‐level CZID results (i.e. the e‐value, aggregate score, read count and reads per million [rPM]) as species information was not available for all taxa. To collapse species to the genus level, we took the minimum, maximum and sums of the e‐value, aggregate score and read counts/rPM, for all species within a genus. To control for potential contamination, CZID uses a “blank” as background to compute a taxon level z‐score, which reflects the likelihood of a taxonomic hit being a contaminant. As these experiments are from many different laboratories using different reagent kits throughout extraction and sequencing, we selected a generic water as the blank sample as it is likely to be analogous to other molecular grade waters used in sample preparation (specifically, “EARLI Novaseq Water Control”).

### 2.4 Generating community count tables

Each CZID taxon report file is produced individually per host sample. Each report file was checked for taxa that matched to non‐microbial sources – such as the host, other invertebrates or plants – and removed when found. These files were then iterated through and non‐host taxon hits were filtered according to the following criteria: (1) read counts were present above 5 reads per million, (2) alignment length was larger than 50 nucleotides, (3) e‐value was below 1*e* − 6, (4) CZID aggregate and z‐scores were above 0 and (5) alignment percent identity was above 90%. This process was run separately for bacteria, eukaryote and viral taxa hit sequences. Though we initially searched for all prokaryotes, only bacteria were detected and we refer to this analysis as such. CZID aligns suspected non‐host reads to both the NCBI nucleotide (NT) and non‐redundant protein (NR) sequence databases. For bacterial and eukaryotic taxa, the above filters were assigned to the taxa hits mapped against the NT database; the viral taxa were assessed against the NR database results. This is necessary as viruses evolve so rapidly that they can fail to map to the NT database but map perfectly well against the more conserved NR database. Viral taxa were analysed at family level, with bacteria and eukaryote taxa at genus level. Results of each host sample were combined into a single counts table per microbial classification (bacteria, eukaryotes and viruses).

### 2.5 Beta diversity (dissimilarity) analyses

Read count tables were further reduced by removing host samples that had fewer than 100 non‐host reads total and microbial taxa that were present in less than 5% of the remaining samples. As sample phylogeny was to be considered in microbial composition, we restricted sample sets to taxa that contained at least four samples to allow for centroid calculation. Host taxa with fewer samples were removed. In the bacterial analysis, this could be done to the level of host tribe, and in the other two analyses, host family.

Beta diversity was calculated with vegan (version 2.6‐4, Dixon, 2003) in R (version 4.2.2, R Core Team, 2020) and its associated functions. Bray–Curtis dissimilarity matrices were calculated for each microbial category using the function avgdist with 10,000 iterations. Other approaches, such as using binary presence/absence matrices, were considered but ultimately decided against. Using this approach, read number can be analogous to either microbial abundance or transcriptional activity or both. In the former case, microbes with relatively increased read number dominate communities by number, and in the latter, their higher level of transcriptional activity has indications for their importance within the community. In either scenario, converting various read abundances above 0 to simply presence (versus absence) would potentially be removing lots of informative data regarding each microbe's significance within its community relative to other members. Rarefaction for each matrix was set to use the lowest number of reads from the smallest sample grouping of sociality – solitary – in order to retain as many samples of that grouping as possible. This read limit was therefore different for each of the three matrices: bacteria *n* = 323, eukaryotes *n* = 171, viruses *n* = 111. Samples with total reads less than this number were discarded. For the virus analysis, two further samples were removed to ensure there were no singletons within social lifestyle, continent or host family factor levels. It was decided that rarefying the reads was the best approach for this analysis in an attempt to reduce the impact of technical effects, such as variation in sequencing depths. Rarefied reads were used to make 10,000 distance matrices, and the final matrix consisted of the average distances computed across these iterations.

Non‐metric multidimensional scaling (NMDS) was used to visualise dissimilarities, computed by metaMDS. To assess whether variables of interest – social lifestyle, phylogeny, location – significantly affected community composition, we performed permutational multivariate analyses (PERMANOVA) using adonis2 with 9999 permutations. Each factor was checked for homogeneity of group dispersion using betadisper to compute average distances around the median, and ANOVA was used to test significance of any difference between groups. Significant differences in dispersion break one of the assumptions of adonis2 and thus factors with heterogeneous dispersal that are implicated as significant drivers of community composition after PERMANOVA should be interpreted with considerable caution.

### 2.6 Predicting microbial communities

We assessed filtered count data for each microbial grouping to determine the prevalence of microbial taxa per host species. The average relative abundance and prevalence of all detected bacterial species were assessed for each tribe of bees. Those at above 50% prevalence and 0.01% average relative abundance per tribe were considered potential members of conserved tribe‐level community, termed here as an “associate” species. Overlaps of bacterial species by sample tribe, family and sociality was also considered. Finally, hosts were checked specifically to see whether they contained any of the core phylotypes found associated with corbiculate bees in previous studies. The prevalence was calculated per tribe for the corbiculates (Apini, Bombini, Meliponini and Euglossini), with non‐corbiculates ordered by sociality.

## 3 RESULTS

### 3.1 Sample selection and CZID pipeline

There were initially 285 bee samples that met the selection requirements for download from the SRA. After filtering out samples that had too few counts after host mapping (in non‐*A. mellifera* samples), the CZID pipeline and further filtering steps, there were 254 samples remaining, containing bee tissue from 4 phylogenetic families (Figure 1), 14 tribes, 18 genera and 45 species from experiments across six continents (Table 1, see Table S1). There were considerably more *Apis* and *Bombus* samples available and included (92 and 65 samples, respectively), and 79.9% of all samples were from the Apidae family, particularly from corbiculate species. A total of 165 samples are considered obligately eusocial, 59 social, and 30 solitary. All samples successfully ran through the CZID pipeline (version 7.1), with 97% passing quality control with more than 50% of input reads.

### 3.2 Detected microbial community

#### 3.2.1 Bacteria

**FIGURE 2:**
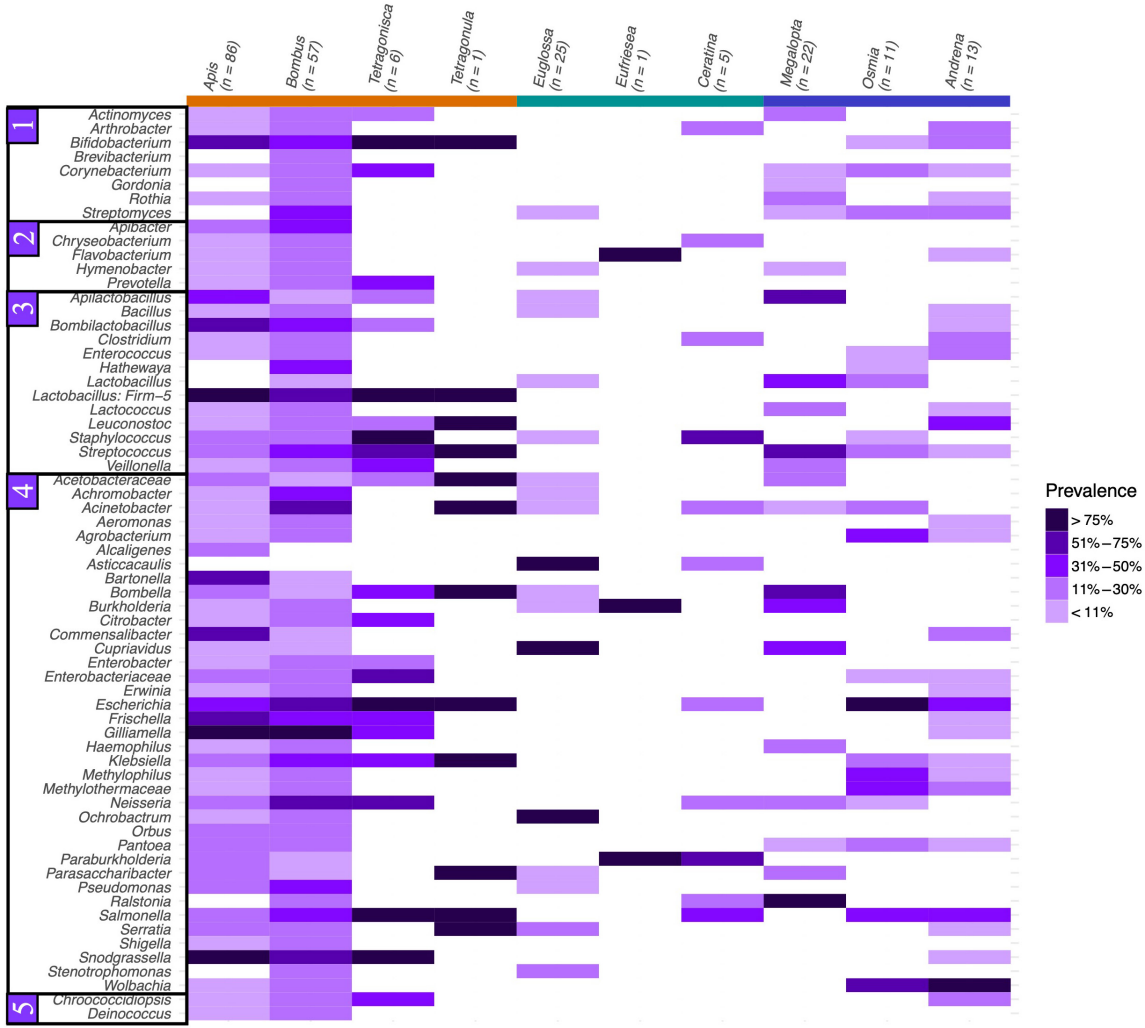
Heatmap of bacterial prevalence in each genus of host. Bacterial taxa are ordered (1) Actinobacteria, (2) Bacteroidota, (3) Firmicutes, (4) Proteobacteria and (5) other. Host genera are coloured by sociality: orange = obligately eusocial, green = social, blue = solitary.

**FIGURE 3:**
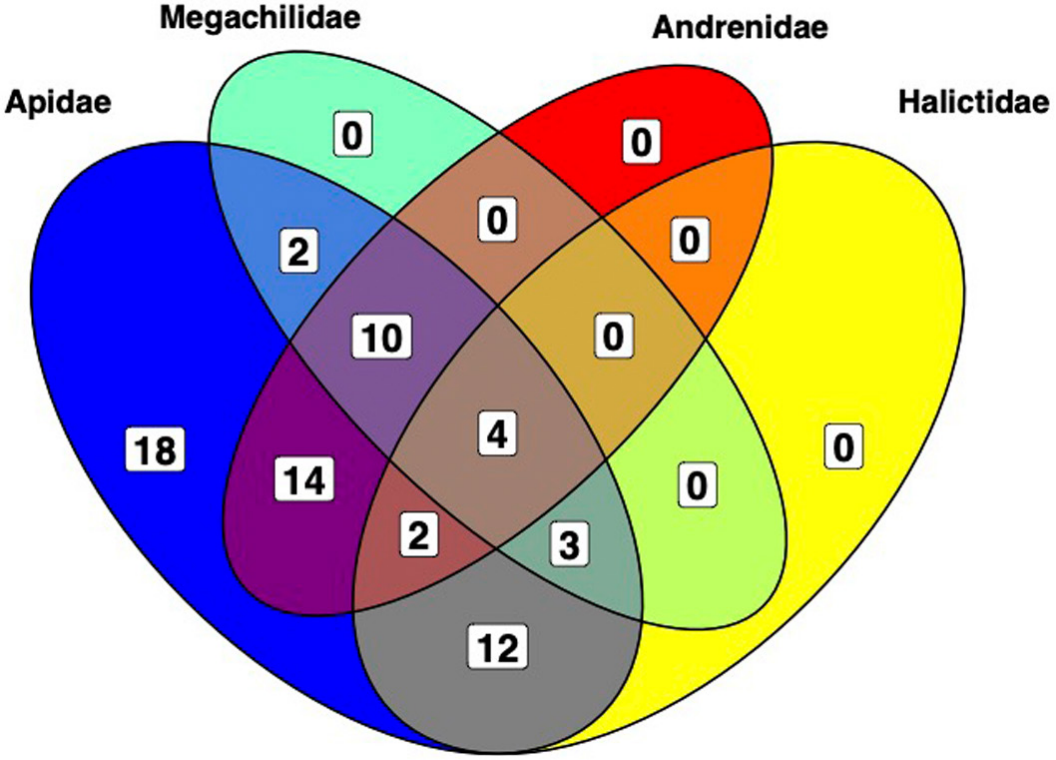
Overlap of bacterial taxa detected in different host families. The only unique taxa are found in Apidae.

There were sufficient reads in 227 samples from 10 bee genera resulting in the detection of 65 bacterial taxa (Figure 2). The most taxa‐rich host family was Apidae, which had unique taxa, while all taxa detected in other families were also present in Apidae (see Figure 3). There were no bacterial taxa found only in solitary hosts, whereas there were 1 and 11 taxa unique to social and obligately eusocial hosts, respectively. The former was *Asticcacaulis*, an associate bacterial taxa of Euglossini samples (Table 2), and the latter consisted of *Lactobacillus*: *Firm‐5*, *Bartonella*, *Apibacter*, *Alcaligenes*, *Brevibacterium*, *Citrobacter*, *Deinoccocus*, *Enterobacter*, *Orbus*, *Prevotella* and *Shigella*. The majority of detected taxa belong to the Proteobacteria phylum.

**TABLE 2:** Associate bacterial taxa found at above 50% prevalence and 0.01% relative abundance per tribe.

Tribe	*n*	Associate taxa
Andrenini	13	*Wolbachia*
Apini	86	Bartonella*, Bifidobacterium*, Bombilactobacillus*, Commensalibacter*, Frischella*, Gilliamella*, Lactobacillus: Firm‐5*, Snodgrassella*
Augochlorini	22	Apilactobacillus*, Bombella*, *Ralstonia*, *Streptococcus*
Bombini	57	*Acinetobacter*, *Escherichia*, Gilliamella*, Lactobacillus: Firm‐5*, Snodgrassella*
Ceratinini	5	*Paraburkholderia*, *Staphylococcus*
Euglossini	26	*Asticcacaulis*, *Cupriavidus*, *Ochrobactrum*
Meliponini	7	Bifidobacterium*, *Escherichia*, Snodgrassella*, *Staphylococcus*
Osmiini	11	*Escherichia*, *Wolbachia*

*Note*: Tribe cells are coloured according to host family: blue for Apidae, red for Andrenidae, yellow for Halictidae, green for Megachilidae. “Corbiculate core” bacterial taxa are indicated with *. Only tribes included in the bacterial dissimilarity matrix were assessed (see Table S1).

#### 3.2.2 Eukaryotic and viral taxa

**FIGURE 4:**
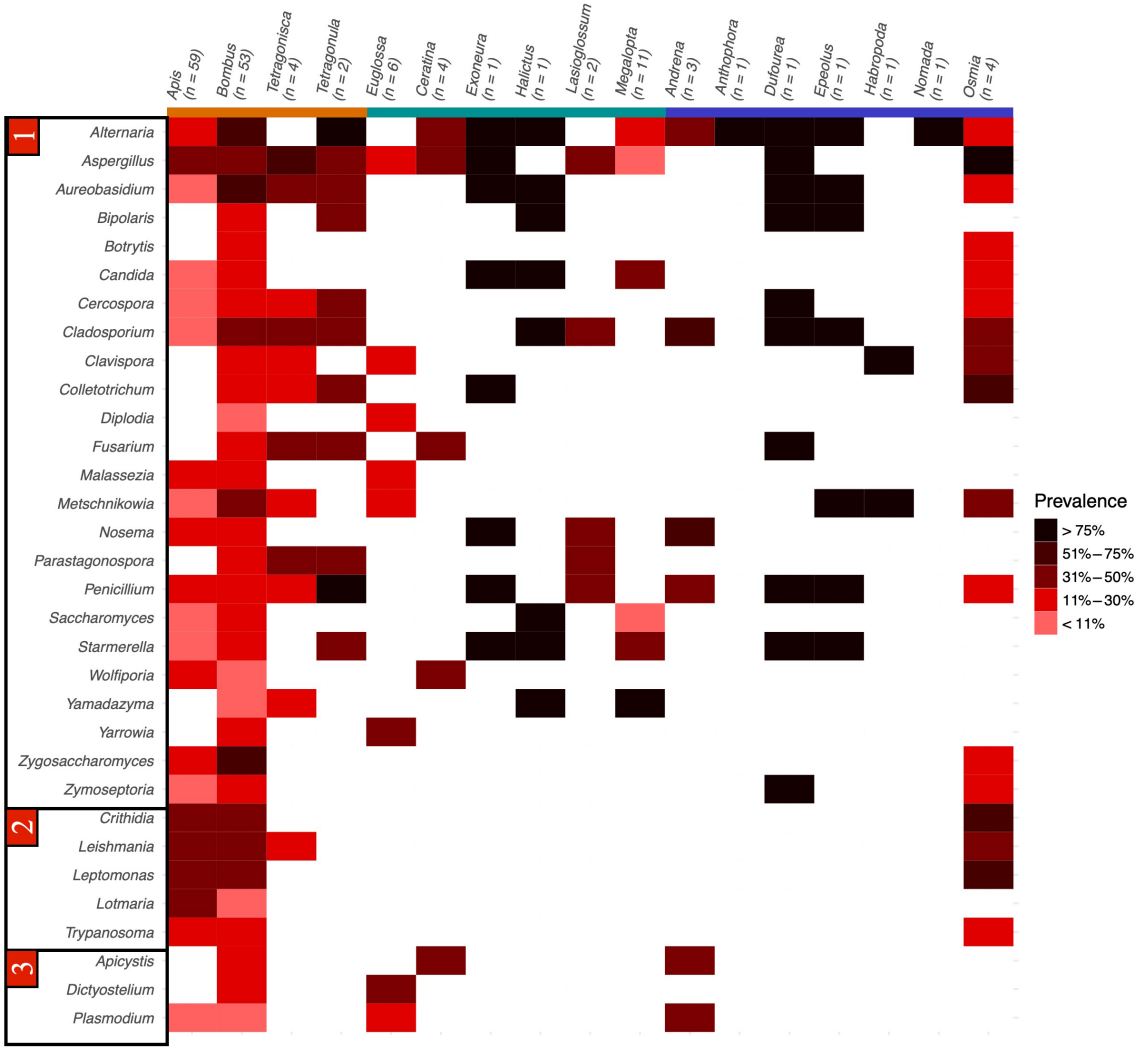
Heatmap of all detected eukaryote taxa and their prevalence in each genus of host samples tested after filtering. Eukaryotic taxa are ordered into (1) fungi, (2) trypanosomatids and (3) other. Host genera are coloured by sociality: orange = obligately eusocial, green = social, blue = solitary.

**FIGURE 5:**
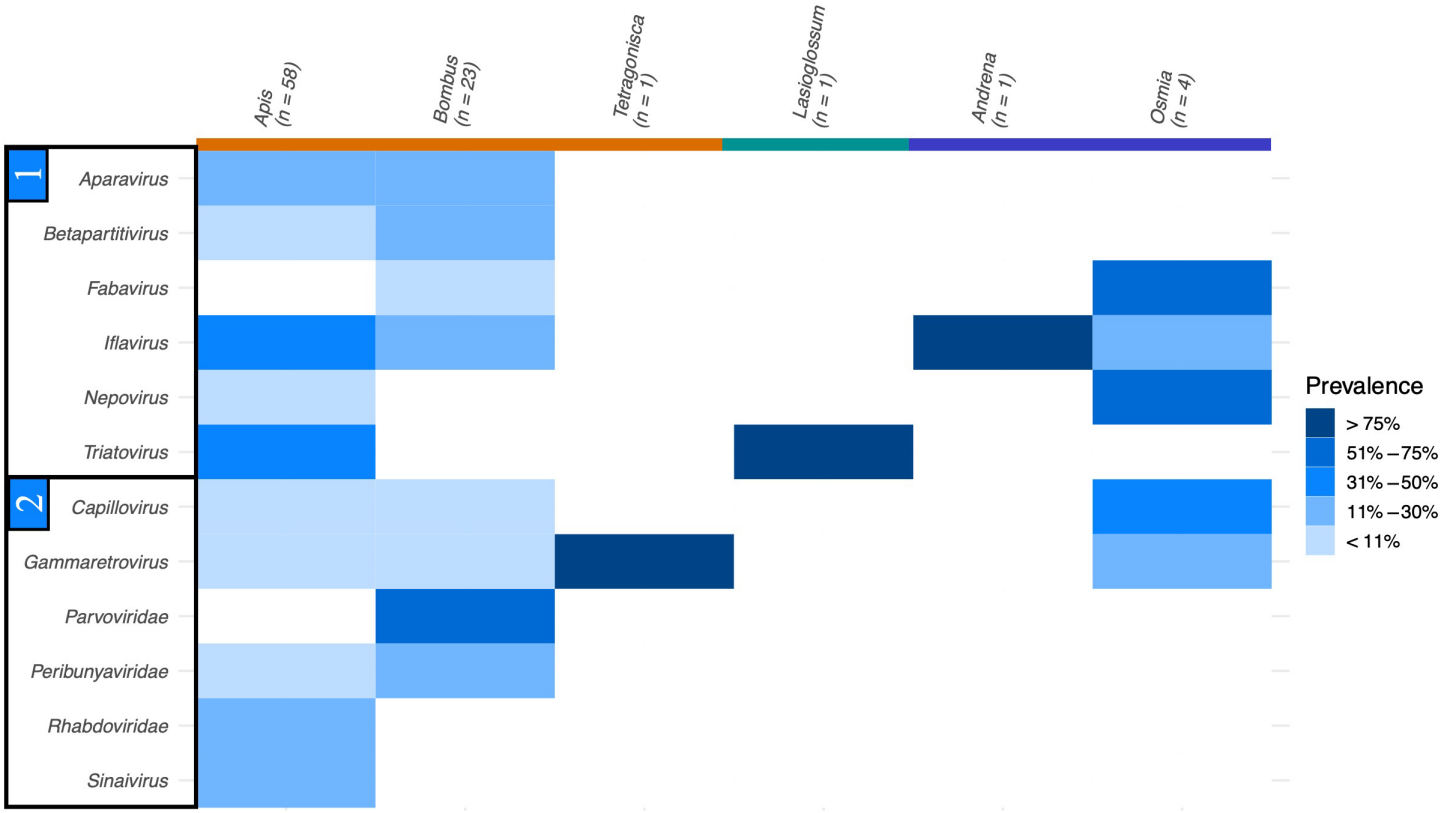
Detected viral prevalence in each host genus that passed data filtering grouped into (1) Pisuviricota and (2) other. Host genera are coloured by sociality: orange = obligately eusocial, green = social, blue = solitary.

There were considerably fewer samples available for determining eukaryote and viral composition after filtering steps. In 158 samples, we identified 32 eukaryotic taxa, including 24 fungi and five genera from the parasitic family Trypanosomatidae (see Figure 4). The two fungal genera *Alternaria* and *Aspergillus* were detected in the majority of species, appearing in 13 and 11 out of 17 genera, respectively. Twelve viral families – six of which from the phylum Pisuviricota – were found across 88 host samples (see Figure 5).

### 3.3 Differences in microbial composition

**FIGURE 6:**
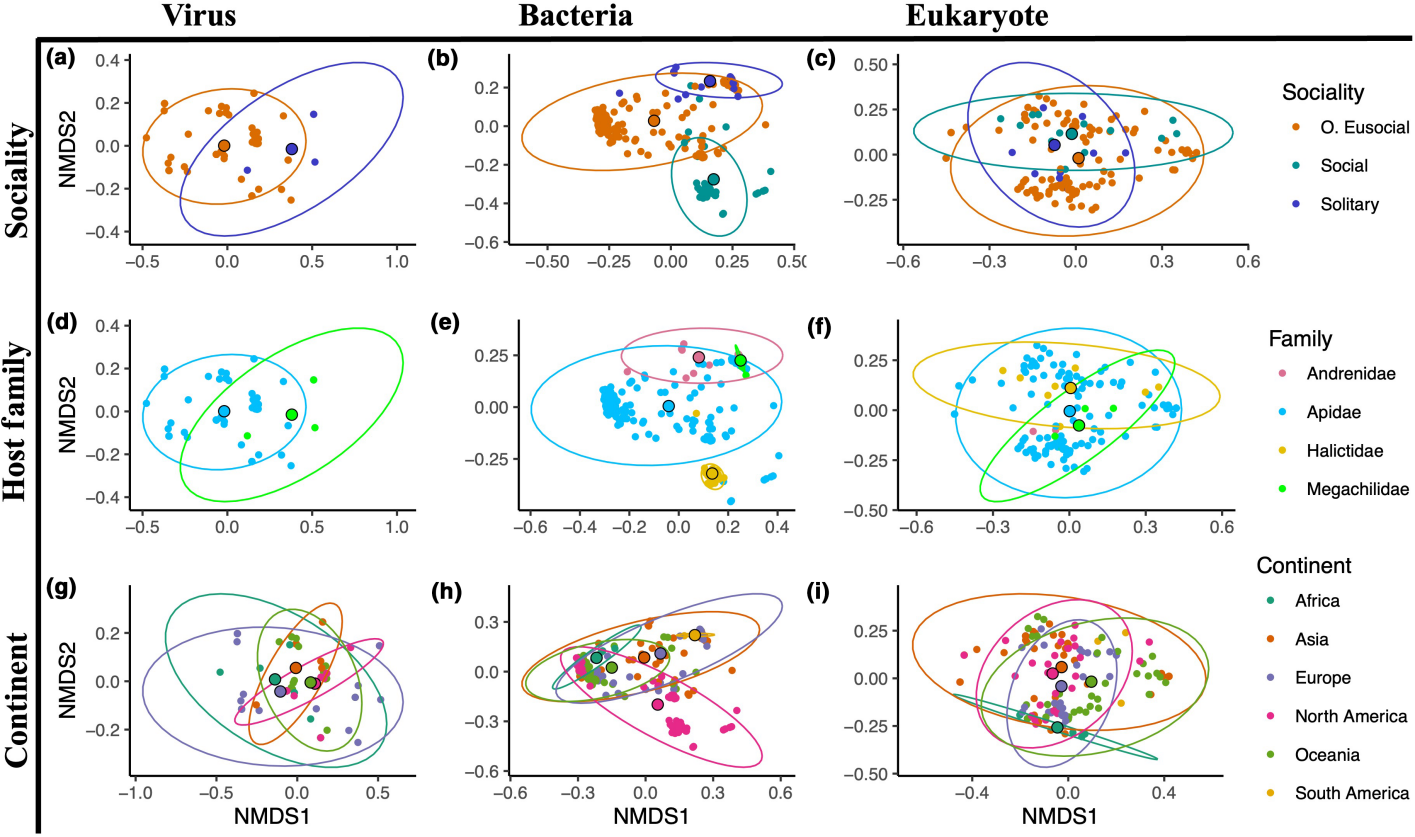
NMDS plots of Bray–Curtis dissimilarity matrices computed separately for virus (column 1), bacterial (column 2), and eukaryote (column 3) reads. Three factors were tested to assess influence on composition: sociality (row 1), host family (row 2) and continent where the samples were collected according to NCBI SRA records (row 3). Centroids for each factor level are shown larger and bordered in black. Axes may differ to accommodate full ellipses.

Sociality significantly influences bacterial composition (Figure 6b), has homogeneous dispersion (see Table S3) and significantly influences the composition of the distance matrix (pseudo‐*F* = 3.1360, *p* = .0002). This was mostly driven by the differences between obligately eusocial and social samples (pairwise PERMANOVA: *p* = .0195, Benjamini–Hochberg correction, see Table S4). Host family and continent (Figure 6e,h) both also significantly affected bacterial composition (pseudo‐*F* = 6.0532, *p* = .0001 and pseudo‐*F* = 2.4902, *p* = .0001, respectively) and are unaffected by heterogeneous dispersion (see Table S4 for pairwise PERMANOVA).

In eukaryotes (Figure 6c,f,i), sociality and continent were statistically significant factors (sociality: pseudo‐*F* = 2.3605, *p* = .0011; continent: pseudo‐*F* = 2.0674, *p* = 1.0282) driving community composition, but both were overdispersed, suggesting caution in interpreting these results (see Table S3). After filtering, there were considerably fewer samples in the viral analysis than were included in either the eukaryotic or bacterial (see Table S1). No social samples survived filtering and sociality and continent factors were completely confounded – all obligately eusocial samples were from the Apidae family, and all Megachilidae samples were solitary. Therefore, when running PERMANOVA, only sociality and location of collection were considered in the model. Neither of which were found to have a significant effect on viral composition of included samples (Figure 6a,d,g, see Table S3).

#### 3.3.1 Tribe–bacterial associates

In the more commonly studied corbiculate tribes – Apini, Bombini and Meliponini – we find at least two previously described “corbiculate core” phylotypes as associate taxa (Table 2). Associate taxa are defined as those found at above 0.01% relative read abundance and in over 50% of the samples in that tribe. All eight of the taxa associated with Apini are included in the core phylotypes. All three of these tribes share an association with *Snodgrassella*, yet there is no overlap between associate taxa of these three and the other corbiculate tribe, Euglossini. *Wolbachia* is an associate of the two solitary tribes included in this analysis.

#### 3.3.2 Corbiculate core taxa

**FIGURE 7:**
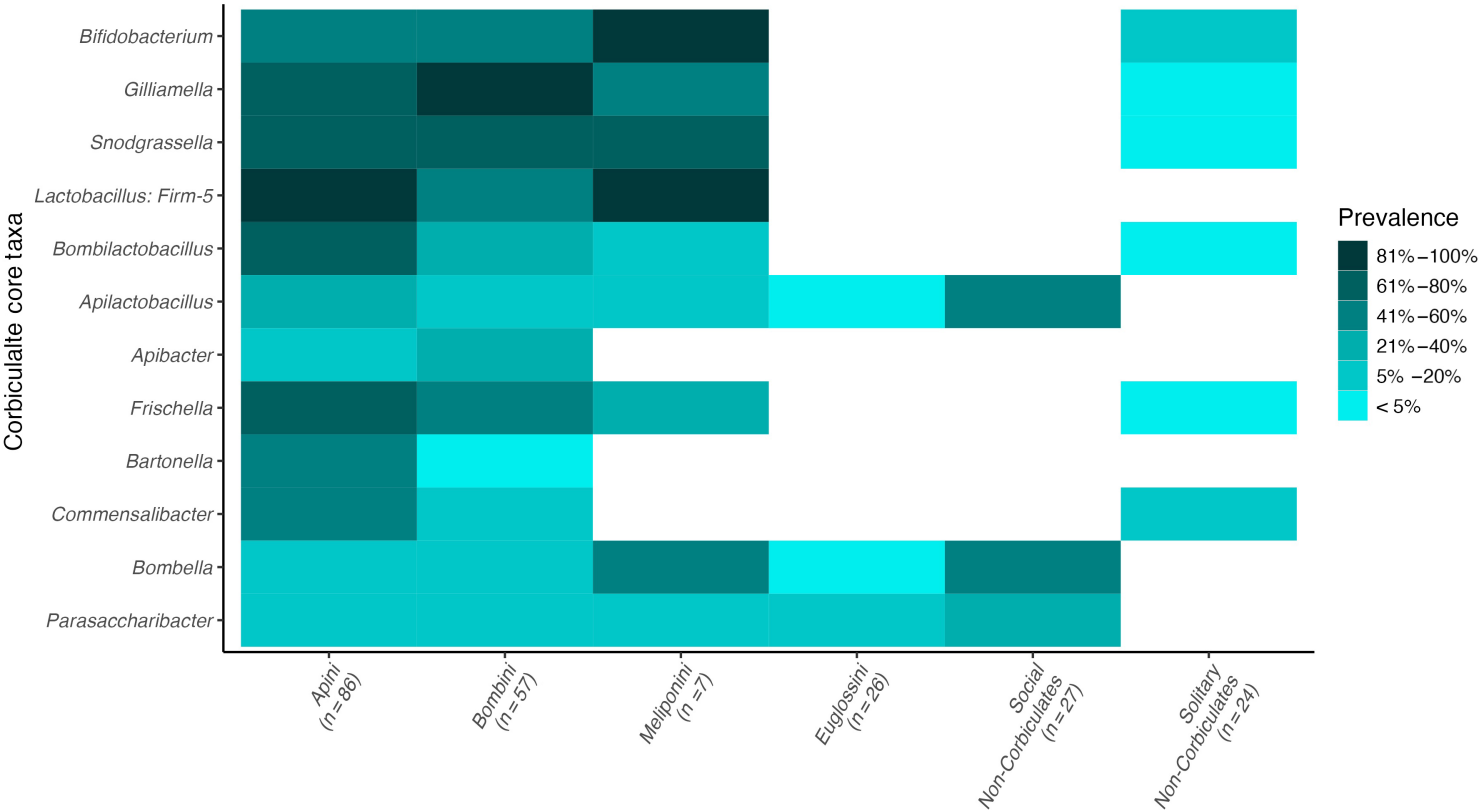
Prevalence of different microbial taxa previously described in the literature as part of the “corbiculate core” bacteria across samples. Core bacterial taxa are mostly absent from Euglossini, a corbiculate tribe. Darker tiles indicate higher prevalence.

We find that the “corbiculate core” bacterial taxa are widely prevalent in the three well‐studied eusocial tribes: Apini, Bombini and Meliponini (Figure 7, see Table S5). This pattern was not repeated in Euglossini, however, where only *Apilactobacillus* was detected at low average relative abundance and prevalence. *Apilactobacillus* was interestingly found at considerable prevalence in social hosts, specifically in the *Megalopta* genus, where it was detected in 15/22 samples. Other bacterial phylotypes were detected in three solitary bee samples: *Bifidobacterium* was detected in one individual *Andrena haemorrhoa* sample (SRR6148367), an individual *Osmia cornuta* (SRR6148371) and in a sample of pooled Andrena individuals of different species (SRR13404633). In the latter, *Gilliamella*, *Snodgrassela*, *Bombilactobacillus* and *Frischella* were also detected. *Bombiscardovia* and *Candidatus Schmidhempelia*, both taxa previously found associated with Bombus bees, were not detected in the analysis after filtering. *Apilactobacillus*, *Bombella* and *Parasaccharibacter* were at considerable prevalence in the social bees. These values are driven largely by *Megalopta* samples (Figure 2).

## 4 DISCUSSION

### 4.1 Bacterial community affected by location, phylogeny and sociality

We find that bacterial communities are significantly affected by social lifestyle, family and collection location of the bee (Figure 6b,e,h, see Table S3). Location and phylogeny have been found to be significant drivers of bee bacterial communities elsewhere, but there is not always consensus on which is more important. While some studies can identify communities to specific subfamilies or even species (Dew et al., 2020; Kwong, Medina, et al., 2017; Kwong & Moran, 2015), others find location to be more informative (Kapheim et al., 2021; Keller et al., 2013; McFrederick et al., 2017; McFrederick & Rehan, 2016, 2019), though often both play a significant role (McFrederick et al., 2012; Shell & Rehan, 2022).

**FIGURE 8:**
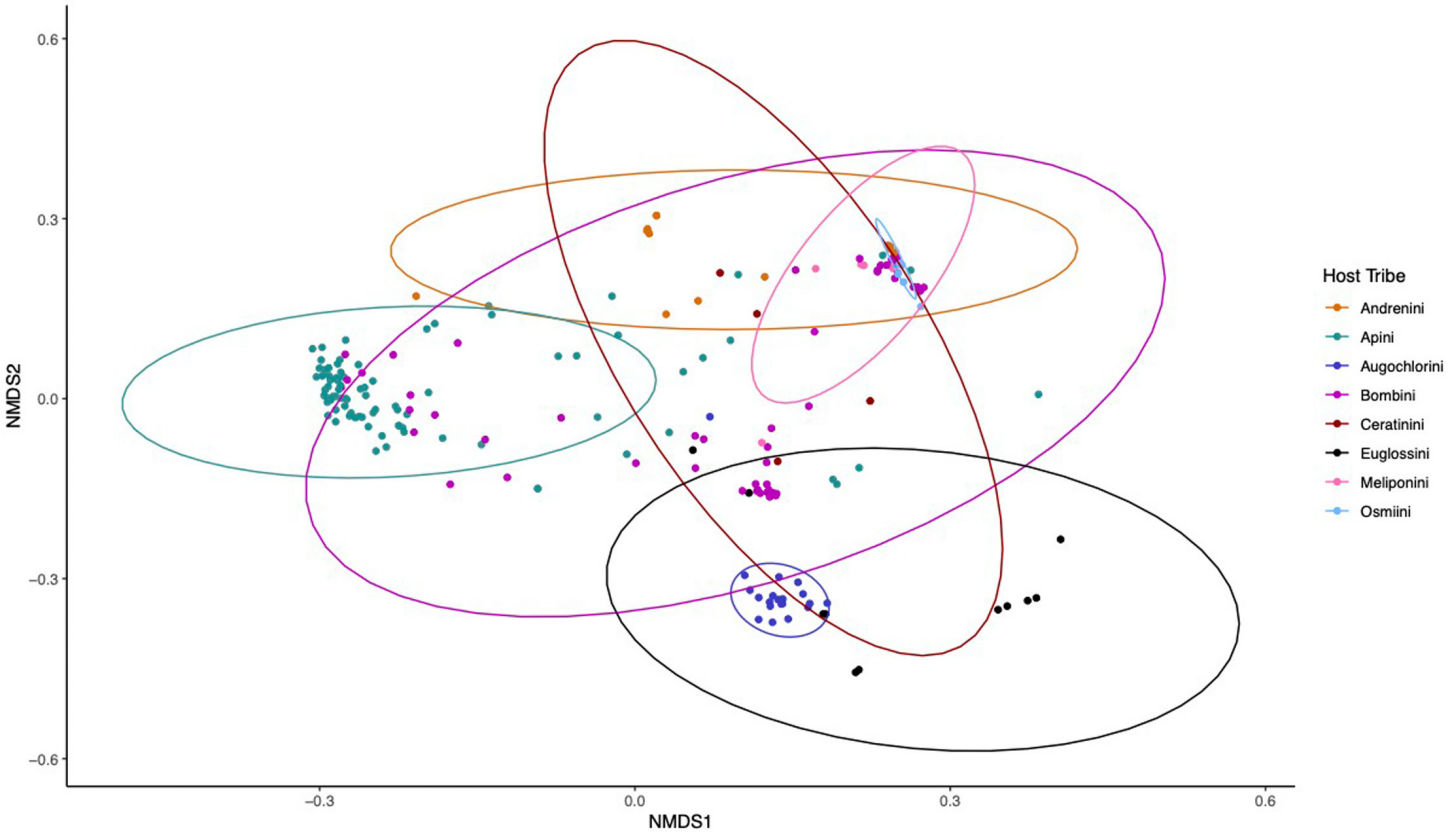
NMDS plot of Bray–Curtis dissimilarity matrix computed for bacterial read counts shows clustering of samples by host tribe. Centroids for each factor level are shown larger and bordered in black. Despite coming from many different projects across several continents, there is a clear cluster of Apini samples.

It is likely that the contribution of each factor is further determined by the social lifestyle of the bee: social living allows for the transmission of symbiont species in eusocial insect societies, where this vertical transmission route allows for coevolution of unique and long‐lasting host–microbe associations (Dietrich et al., 2014; Lombardo, 2008; Sanders et al., 2014; Zhang & Zheng, 2022). Solitary animals, on the other hand, are likely to have less stable communities that are largely acquired from the immediate environment (Voulgari‐Kokota et al., 2019). We see this in some of the obligately eusocial samples: when the bacterial community NMDS plots are clustered by tribe, there is a clear group of Apini samples to the left of the NMDS1 (Figure 8), despite the fact that these samples came from 11 different countries across five continents (Table 1, see Table S1). The limited availability of samples from solitary species does, in turn, somewhat limit the ability to untangle the microbial community composition of solitary bees. Specifically, the samples we analysed from solitary tribes Andrenini and Osmiini were primarily derived from a handful of studies (see Table S1). In cases like these, factors such as phylogeny, sociality and collection location are entangled not only with each other but also with other potentially influential technical factors, such as sample processing. Furthermore, experimental work would be necessary to unravel these factors and ascertain the genuine determinants of microbial community composition.

If solitary species have microbiomes that are predominantly environmentally acquired and lack the consistent vertical transmission of eusocial bees, then they should be more variable and show greater dispersion around the median than more social groups. We do find this (see Table S3), but the differences in variance is small and non‐significant. Future work that includes more solitary samples would be able to better test whether solitary species have more variable microbial communities than the well characterised and more strongly vertically transmitted social microbiomes.

### 4.2 Social lifestyle impacts number and type of associate taxa

Tribes made of obligately eusocial species have the most associate microbe species in this analysis (Table 2), with Apini, Bombini and Meliponini being associated with eight, five and four bacterial genera, respectively. Of these, at least two bacterial taxa were from the identified “corbiculate core” per tribe (Figure 7). This again lends weight to the hypothesis that vertical transmission leads to more stable communities in the social bees, allowing for the establishment of multiple fixed associations. We also detect more associated bacterial genera with increasing number of samples (Table 2), though it should be mentioned that Meliponini has double the identified associate taxa from fairly few samples relative to the solitary tribes.

We find *Wolbachia* associated with the two solitary tribes, Andrenini and Osmiini. *Wolbachia* reads were also detected at low prevalence in Apini and Bombini (Figure 2), but at comparably low average relative abundance (see Table S6). *Wolbachia* is an extremely successful insect endosymbiont, estimated to be present in as much as 52% of all insect species (Weinert et al., 2015). This endosymbiont is capable of manipulating the reproduction of its host in order to spread throughout populations, most famously by inducing cytoplasmic incompatibility (Bourtzis et al., 1996; Werren et al., 2008), and has been proposed to be a potential factor behind *Andrena* diversification (McLaughlin et al., 2023). In the bees, increased *Wolbachia* prevalence and diversity associated with solitary over social species has been described before (De Ramalho et al., 2021; Gerth et al., 2011, 2015; Saeed & White, 2015), though the reasons for this remain speculative. As *Wolbachia* is maternally inherited, it may be that obligately eusocial societies that consist of many sterile or reproductively constrained females would be considered an evolutionary dead‐end for the symbiont, if it were not established that *Wolbachia* persists in high prevalence in a number of eusocial ant species (De Ramalho et al., 2018, 2021; Russell, 2012). It has been previously proposed that this disparity in *Wolbachia* presence between social and solitary bees occurs either due to solitary individuals having a greater number of interactions with other potentially infected taxa, or that social species have a more limited number of ecological environments within which they forage and live (De Ramalho et al., 2021).

We postulate that perhaps this disparity is more to do with obligately eusocial bees having these evolutionary long‐term and stable host–microbe relationships that solitary insects are not able to achieve with their relative lack of social and inter‐generational interaction. Perhaps *Wolbachia* fails to persist in social bees because the established community protects against it, at least in the case of the most social corbiculates. Many features of the social bee core microbes already identified could play a part, such as priming the host immune system (Horak et al., 2020; Kwong, Mancenido, & Moran, 2017; Lang et al., 2022; Näpflin & Schmid‐Hempel, 2016) or the occurrence of direct antagonistic microbe–invader interactions (Dyrhage et al., 2022; Endo et al., 2012; Endo & Salminen, 2013; Koch & Schmid‐Hempel, 2012; Steele et al., 2017; Vásquez et al., 2012). Solitary bees – such as *Andrena* species (McLaughlin et al., 2023) – missing these interconnected communities would therefore lack the protection they confer and may become vulnerable to *Wolbachia* driven reproductive manipulation.

Interestingly another, at least partially, intracellular microbe genus, *Sodalis*, was found to be less abundant in social bees compared to solitary relatives (Rubin et al., 2018). This analysis considered Halictid bees, a lineage where eusociality is considered a relatively recent evolutionary development (Brady et al., 2006). The authors hypothesised that they were detecting the clearance of the microbe from social lineages – including between variants of the socially polymorphic species *Lasioglossum albipes* – and proposed that there was something about increased social contact that was driving the reduction.

A final consideration is that, though often parasitic, *Wolbachia* can be advantageous to hosts conferring nutritional or fecundity benefits (Andersen et al., 2012; Cheng et al., 2019; Singh & Linksvayer, 2020) or resistance to viral or parasitic infection (Bian et al., 2010; Cogni et al., 2021; Duplouy et al., 2015; Pimentel et al., 2021; Van Den Hurk et al., 2012). Future work testing whether *Wolbachia* are beneficial or virulent symbionts in solitary bee species would be most welcome.

### 4.3 “Corbiculate core” microbes may be specific to the obligately eusocial clades

Despite being an important group of pollinators, the orchid bees (Euglossini) remain the least studied group of corbiculate bees and, at the time this paper was written, their microbiomes were undescribed. Two of the three orchid bee species included in this analysis – *Euglossa dilemma* and *E. viridissima* – exhibit some primitively eusocial behaviour, where a mother foundress and a subordinate daughter (sometimes two) administer brood care (Cocom Pech et al., 2008; Saleh et al., 2022). In these instances, there is the increased opportunity of vertically transmitted microbes becoming established across generations, although the fact that some daughters leave the nest after eclosure would suggest these relationships could be less stable than those in obligately eusocial corbiculates. In this analysis – looking at 26 orchid bee samples – we found three Euglossini associate microbial taxa – *Asticcacaulis*, *Cupriavidus* and *Ochrobactrum* – none of which being a member of the previously described corbiculate core (Kwong, Medina, et al., 2017).

Perhaps this “corbiculate core” community is a misnomer, and that what had been previously described were communities shared only between the obligately eusocial corbiculates. There are phylogenetic implications of this insight. While the phylogeny of the corbiculates has historically been controversial, most analyses today place Euglossini as the outgroup to the other three tribes (Bossert et al., 2019; Engel & Rasmussen, 2021). Potentially, then, this core microbiome shared between Apini, Bombini and Meliponini may be as ancient as their last common ancestor (LCA) and was composed after the split between the orchid bees and other corbiculates. It could therefore be argued that this LCA would have likely been obligately eusocial, allowing these bacterial communities to establish stably enough to be passed on to three different lineages through ∼55 million years of host diversification (Peters et al., 2017).

It is worth noting that our Euglossine sample size was limited (*n* = 26), and mostly consisted of *Euglossa* samples. Larger sample sizes and more species may reveal a more complicated picture of Euglossine species presenting with some or all of the “corbiculate core” microbes. However, the sample size for the Meliponini bees in this analysis was considerably smaller (*n* = 7), and yet, this core community was detectable. As this study was under review, Kueneman et al., 2023, also failed to detect a stable set of core microbes among orchid bee species, lending further weight to these findings. It should be noted, however, that their findings also suggested that the relationship between the “corbiculate core” and stingless bees also may not be as strong as previously theorised, suggesting a complicated picture that requires further investigation.

### 4.4 Bacteria with anti‐pathogen potential persist across bee taxa

Though the “corbiculate core” community was not similar between Euglossini and the classic corbiculate tribes, there were other shared microbial taxa. *Apilactobacillus* and *Bombella*/*Parasaccharibacter* – likely to actually be one genus (Smith et al., 2021) and referred to hereafter as *Bombella* – were detected in orchid bees and at considerable prevalence in *Megalopta* (Figure 7, see Table S5). Similarly, both *Apilactobacillus* and *Bombella* were detected in five and six of the ten species included in the bacterial analysis, respectively (Figure 2), though not in either of the solitary genera.

One of the reasons why these two bacterial groups are so successful at establishing in such diverse bee taxa may be their roles as anti‐pathogen symbionts. *Bombella*, for example, has anti‐fungal properties (Miller et al., 2021) and is found frequently in honeybee larvae and food stores, two components of the colony which are especially vulnerable to fungal infection (Anderson et al., 2014). This would also be an advantage to any host that stores pollen, and could help explain its presence in most of the social species in this analysis. *Apilactobacillus* increases individual resistance to a number of pathogens including *Paenibacillus larvae* (American foulbrood, Butler et al., 2013; Forsgren et al., 2010; Kačániová et al., 2020; Kiran et al., 2022), the microsporidian *Nosema* (Arredondo et al., 2018), fungal infection (Iorizzo et al., 2020) and *Melissococcus plutonius* (European foulbrood, Endo et al., 2012; Endo & Salminen, 2013; Vásquez et al., 2012; Zendo et al., 2020). It is also prevalent in the floral environment, suggesting an intuitive route for transmission between different bee species visiting the same flowers (Anderson et al., 2013; Tamarit et al., 2015).

Many other members of the “corbiculate core” community may also confer resistance to common bee pathogens. *Snodgrassella* increases honeybee resistance to *Serratia marcescens* infection (Horak et al., 2020); in bumblebees, *Gilliamella* and *Apibacter* suppress trypanosomatid *Crithidia* species (Cariveau et al., 2014; Mockler et al., 2018); and members of *Lactobacillus: Firm‐5* inhibit *P. larvae* and *M. plutonius* growth (Killer et al., 2014) and *C. bombi* infection in bumblebees (Mockler et al., 2018). The mechanisms of this protection could be host moderated, for example by increasing the expression of immune‐associated genes (Horak et al., 2020; Kwong, Mancenido, & Moran, 2017), allowing for immune priming (Milutinović et al., 2016; Sadd & Schmid‐Hempel, 2006), or symbiont moderated, for example by creating a physical barrier to pathogen colonisation (Kwong & Moran, 2013; Martinson et al., 2012) or producing anti‐pathogen molecules (Dyrhage et al., 2022; Endo et al., 2012; Endo & Salminen, 2013; Koch & Schmid‐Hempel, 2012; Steele et al., 2017; Vásquez et al., 2012).

The preponderance of anti‐pathogen effects by bee‐associated microbes may be linked to the immune gene architecture of bees. When the honeybee genome was first sequenced (Honeybee Genome Sequencing Consortium & others, 2006), one of the curious features was the relative lack of immune genes compared to other insect models (Evans et al., 2006). This was surprising for the honeybee, a eusocial insect that lives in societies of thousands of genetically similar individuals that are thus vulnerable to pathogen spread. Initially, this disparity was explained by the unique benefits of social immunity – a suite of behaviours that social animals use to help prevent and slow disease transmission, such as allogrooming and expulsion of the sick (Cremer et al., 2007, 2018; Dolezal & Toth, 2014; Wilson‐Rich et al., 2009) – leading to relaxed selection on individual immunity and, eventually, gene loss. However, as more bee genomes became available, it became clear that this depauperate immune gene repertoire predated bee sociality (Barribeau et al., 2015).

This restricted immune genetic architecture could perhaps be why *Apilactobacillus* is often found outside of the classic corbiculate bees, as is found in this analysis and elsewhere (Handy et al., 2022). In *Apilactobacillus kunkeei*, a plasmid causes one strain's antibacterial effects against *M. plutonius* (Endo & Salminen, 2013; Zendo et al., 2020). Upon further investigation, more plasmids putatively encoding antibiotic compounds were discovered in other strains (Dyrhage et al., 2022). Similarly, *Apilactobacillus kunkeei* is usually found as multiple strains within hosts where transfer of mobile genetic elements is common (Tamarit et al., 2015). These features allow for the rapid evolution of *Apilactobacillus* and may represent an example of an extended immune phenotype where the genetic potential of *Apilactobacillus* – and, perhaps, many other strains of bee‐associated taxa – compensates for the relatively restricted host immune genetic potential. It is also possible that similar extended immunity phenotypes are occurring in the solitary bees – for example, the putative antiviral capability of *Wolbachia* – but these would require further investigation. It is likely that the relative lack of social contact‐driven vertical transmission within solitary species means that such relationships, when they occur, may be much more taxon‐specific and less permanent than what has been found in more social bees. Perhaps there are other species that, like *Wolbachia*, have evolved mechanisms to ensure high‐fidelity vertical transmission without the need for consistent social interactions.

### 4.5 Mining RNA‐Seq samples recapitulates experimental findings in obligately eusocial corbiculates

The composition of the “corbiculate core” microbiome has been well characterised (P. Engel et al., 2012; Engel & Moran, 2013; Koch et al., 2013; Koch & Schmid‐Hempel, 2011; Kwong, Mancenido, & Moran, 2017; Kwong & Moran, 2016; Moran et al., 2012), making it a good yardstick against which we could assess the efficacy of using this pipeline to detect microbial communities. Out of the 14 microbes we opted to include as members of this core set, 12 were detected – the supposedly *Bombus*‐specific *Bombiscardovia* and *Candidatus Schmidhempelia* were not detected in any samples after filtering. Having several samples per host taxa obviously improves the reliability of any detected compositions or associations, though it should be reiterated that the core microbes were recapitulated in Meliponini samples despite the relative lack of individual samples (Figure 7). We also detected the disparity in *Wolbachia* presence and abundance between social and solitary bees (Table 2), as previously described (De Ramalho et al., 2021; Gerth et al., 2011, 2015; Saeed & White, 2015). Further to this, our findings regarding the lack of “corbiculate core” microbes in Euglossini species has been reported since the analysis was undertaken (Kueneman et al., 2023).

While useful, this approach does have limitations. Firstly, we did not reach 100% detection of predicted microbes, and, thus, some individual microbes are potentially being missed. The majority of samples were prepared using poly‐A enrichment as part of their library preparation (see Table S1), which significantly reduces the level of non‐eukaryotic RNA in the sample (Cui et al., 2010). This does limit our ability to comment on any differences in absolute abundances, and the likelihood that species either found in vivo at low abundances or else that are relatively transcriptionally inactive are missed by this approach cannot be overlooked. Further to this, the fact that we were limited to using only one “blank” for the pipeline run – and that we had no access to any reagents from any of these archived experiments – means that, though many human/reagent contaminants have been removed/reduced from our findings, we cannot ideally control for contamination.

However, despite these considerations, this approach has consistently detected the key bacterial taxa that are expected in species already described throughout the literature and thus indicates the potential of using this method – or others like it using transcriptomic data – to estimate community compositions. It is possible that utilising transcriptomic data, particularly from libraries that remain unaltered to minimise non‐eukaryotic reads, may provide deeper insights than DNA sequencing. Taxa with high read abundances could serve as indicators of more metabolically active and dominant community members, which has significant implications for host health and functionality.

In the course of this investigation, we have uncovered several intriguing avenues for future research based on the analysis of existing sequencing data. Furthermore, there remains untapped potential within this dataset, particularly in the realm of assessing the functional distinctions among detected microbial reads. For example, do all Euglossini species lack the classic “corbiculate core” shared among its relatives (Figure 7)? Would the pattern of increasing numbers of bacterial associates with increasing social complexity hold when more solitary species are included (Table 2)? How do other factors such as seasonality or sex affect these patterns? What are the phylogenetic relationships of bacterial species with many hosts such as *Apilactobacillus*? Do obligately eusocial hosts with long‐standing microbial relationships act as evolutionary reservoirs for bee symbionts?

### 4.6 Conclusion

By leveraging existing RNA sequencing datasets, we were able to test whether microbial communities are affected by social structure, geography or phylogeny. We found that bacterial community composition is significantly affected by the social lifestyle, collection location and phylogeny of the host (Figures 6, 8). In the eukaryotic and viral analyses, however, we failed to detect any factor contributing to community composition that was not affected by heterogeneous dispersion (see Table S3). It appears that as the complexity of social lifestyle increases, so too does the number of bacterial associates (Table 2). This may be expected as prolonged social contact between host generations allows for more reliable vertical transmission and coevolution of host and symbiont, but including more non‐obligately eusocial samples will be necessary to test this hypothesis. We also provide an initial description of the microbial community of the Euglossine bees, species that do not align with the regimented core microbes of their sister corbiculates (Figure 7). The anti‐pathogen potential of microbial symbionts is massive, which may be how bees compensate for their own restricted immune gene arsenal. This work has highlighted many avenues that represent promising lines of future research and the need to further investigate species of varying social lifestyles outside of the classic corbiculate bees. Hopefully, further work into the complicated, genetically mobile world of bee symbionts will further illuminate host–microbe complexities and their role in optimising bee health.

## AUTHOR CONTRIBUTIONS

**Lauren Mee:** Conceptualization (equal); formal analysis (lead); investigation (lead); methodology (lead); project administration (equal); visualization (lead); writing – original draft (lead). **Seth M. Barribeau:** Conceptualization (equal); funding acquisition (lead); project administration (equal); supervision (lead); writing – review and editing (lead).

## Supporting information


Table S1.

Table S2.

Table S3.

Table S4.

Table S5.

Table S6.

## Data Availability

The complete bioinformatic and analysis pipeline with accompanying scripts, taxon reports, count tables, distance matrices and directions are available from: https://github.com/LMee17/AnthoMicroComp/.
